# Case Report: Spinal arteriovenous fistula and vertebral angiomatosis in a cat—the role of imaging within the vascular malformation spectrum

**DOI:** 10.3389/fvets.2026.1862231

**Published:** 2026-06-16

**Authors:** Sooyoung Oh, Seoungyob Ahn, Kichang Lee, Hakyoung Yoon

**Affiliations:** 1 Department of Veterinary Medical Imaging, College of Veterinary Medicine, Jeonbuk National University, Iksan-si, Jeollabuk-do, Republic of Korea; 2 VIP Animal Medical Center, Seoul, Republic of Korea

**Keywords:** case report, feline, magnetic resonance imaging, spinal arteriovenous fistula, vertebral angiomatosis, computed tomography

## Abstract

Spinal arteriovenous fistula (SAVF) and vertebral angiomatosis are rare vascular malformations reported in cats, and their clinical and imaging manifestations may partially overlap. This report describes a T11 vertebral lesion in a 2-year-old spayed female cat that presented with non-ambulatory paraparesis and difficulty in urination and defecation. Computed tomography (CT) and magnetic resonance imaging (MRI) revealed an expansile osseous lesion centered on the left-sided lamina, pedicle, and vertebral body of T11, resulting in extradural spinal cord compression. Notably, the dilated venous structures or obvious arteriovenous shunts commonly described in previously reported feline SAVF cases were not identified on imaging. Following surgical decompression, two separate histopathological evaluations yielded different diagnoses: arteriovenous fistula with osseous proliferation and bone remodeling, and capillary-type vertebral angiomatosis. However, these findings may not be mutually exclusive; rather, they can be interpreted as emphasizing different aspects within a vascular malformation spectrum in which the two entities share a common vascular pathophysiology. This case demonstrates that a feline spinal vascular lesion presenting with vertebral osseous proliferation may show imaging features consistent with both vertebral angiomatosis and SAVF. It also suggests that imaging plays a crucial role in evaluating the predominant pathophysiologic mechanism responsible for neurological signs and in guiding therapeutic decision-making.

## 1 Introduction

Spinal arteriovenous fistula (SAVF) and vertebral angiomatosis are both rarely reported conditions in cats. These disorders typically affect young cats and most frequently involve the thoracic vertebrae. Clinical manifestations are often similar and may include paraparesis, spinal pain, and urinary or fecal dysfunction (1–10).

Angiomatosis is histopathologically characterized by proliferation of well-differentiated vascular structures in both human and veterinary literature (11, 12), and when occurring in the vertebra, is commonly associated with vertebral proliferation and extradural spinal cord compression in cats (1–8). In contrast, an arteriovenous fistula (AVF) is defined by a direct communication between the arterial and venous systems bypassing the capillary bed (11, 13), resulting in altered spinal hemodynamics (14, 15). Both conditions are classified within the broader category of vascular malformations (11, 16, 17) and have been reported to share overlapping histopathologic features (11, 18).

In previously reported feline SAVF cases, diagnostic imaging has commonly demonstrated dilated venous structures and evidence of abnormal arteriovenous shunt (10). By comparison, vertebral angiomatosis is typically characterized by thickening or mass-like lesions of the vertebra (1–9). However, recent veterinary and human reports suggest that SAVF accompanied by reactive osseous proliferation may present as an expansile vertebral lesion resembling angiomatosis (19–22).

To the best of our knowledge, no feline cases exhibiting concurrent imaging and histopathologic features of both SAVF and vertebral angiomatosis have been previously described in the literature. Here, we report such a case and discuss imaging-based assessment of the predominant pathophysiologic mechanism within the vascular malformation spectrum.

## 2 Case description

A 2-year-old spayed female Scottish Fold cat weighing 4.7 kg was presented with a 2-week history of decreased activity and progressive hindlimb weakness, which advanced to non-ambulatory paraparesis accompanied by dysuria and dyschezia. Physical examination, complete blood count, serum biochemistry and urinalysis revealed no abnormalities related to the presenting neurologic signs. Both feline leukemia virus and feline immunodeficiency virus tests were negative.

Neurological examination revealed thoracolumbar spinal pain, diminished postural reactions in the pelvic limbs, and non-ambulatory paraparesis, localizing the lesion to the T3–L3 spinal cord segments. No definitive evidence of thrombus indicative of feline arterial thromboembolism was identified on abdominal ultrasonography. However, a subtle and poorly marginated increase in opacity was identified at the T11 vertebral foramen on plain radiography (Figure 1A).

**Figure 1:**
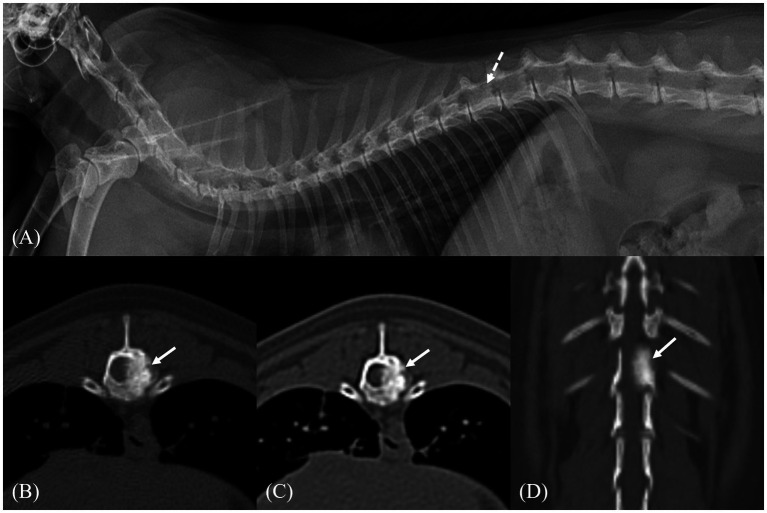
**(A)** Right lateral thoracic radiograph showing a faint, poorly marginated increased opacity at the level of the T11 vertebral canal (dotted arrow). **(B)** Transverse pre-contrast CT, **(C)** transverse post-contrast CT, and **(D)** dorsal pre-contrast CT images. An irregularly marginated, bone-attenuated mass-like lesion involving the left lamina, pedicle, and vertebral body of T11 is observed (arrow), resulting in narrowing of the vertebral canal. On post-contrast images, the lesion shows an increase of more than 200 Hounsfield units (HU), with no significant osteolysis. On the dorsal image **(D)**, the lesion extends along the entire length of the T11 vertebra.

CT demonstrated an irregular osseous proliferative lesion involving the left-sided lamina, pedicle, and vertebral body of T11, forming a mass-like structure (Figures 1B–D). No evidence of osteolysis was identified. Because the lesion contained osseous components, direct visual assessment of intralesional contrast enhancement was limited. However, quantitative comparison of Hounsfield unit (HU) values between pre- and post-contrast images demonstrated an increase of more than 200 HU at all measured sites within the lesion, suggesting contrast agent influx. MRI further characterized the lesion at the level of T11, demonstrating hypointense signal intensity on both T1- and T2-weighted images and resulting in up to 77% spinal cord compression (Figure 2). The compression ratio was calculated as the percentage of the spinal cord cross-sectional area at the maximal compression site relative to a cranial normal segment (23). In addition, the lesion was considered extradural because it was continuous with the left T11 pedicle and vertebral body, with rightward displacement of the epidural fat, left subarachnoid space, and spinal cord. An intradural component was considered less likely because the subarachnoid space was displaced while maintaining its contour, without focal expansion along the cranial and/or caudal margins of the lesion, which is typically seen with an intradural mass (24).

**Figure 2:**
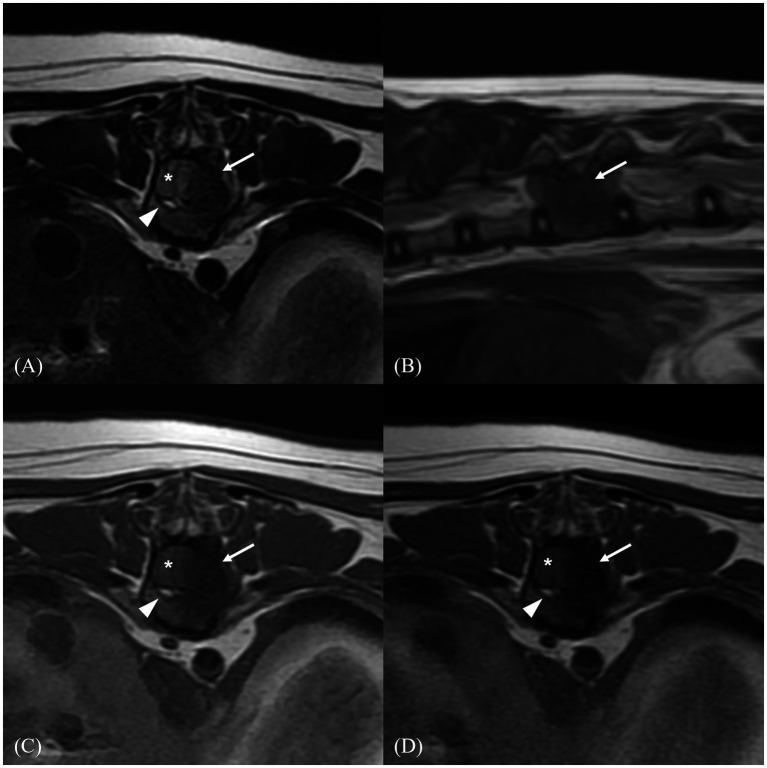
**(A)** Transverse T2-weighted, **(B)** parasagittal T2-weighted, **(C)** transverse T1-weighted pre-contrast, and **(D)** transverse T1-weighted post-contrast MRI images at the level of T11 demonstrate a left-sided extradural lesion within the vertebral canal (arrow). The lesion is hypointense on both T1- and T2-weighted images. It results in rightward displacement of the subarachnoid space and epidural fat (arrowhead), and the spinal cord (asterisk).

A paramedian incision was made following a left-sided approach. After incising the fascia lateral to the spinous process, the epaxial muscles were retracted ventrally to expose the left pedicle of T11. Grossly, the exposed bone exhibited a rougher surface than the adjacent vertebrae (Figure 3). Samples of the abnormal bone tissue were obtained intraoperatively by excisional sampling and submitted for histopathological evaluation; the remaining lesion was then excised using a high-speed burr. Partial corpectomy was then performed to decompress the spinal cord, and autologous fat harvested from subcutaneous tissue was applied over the decompressed area.

**Figure 3:**
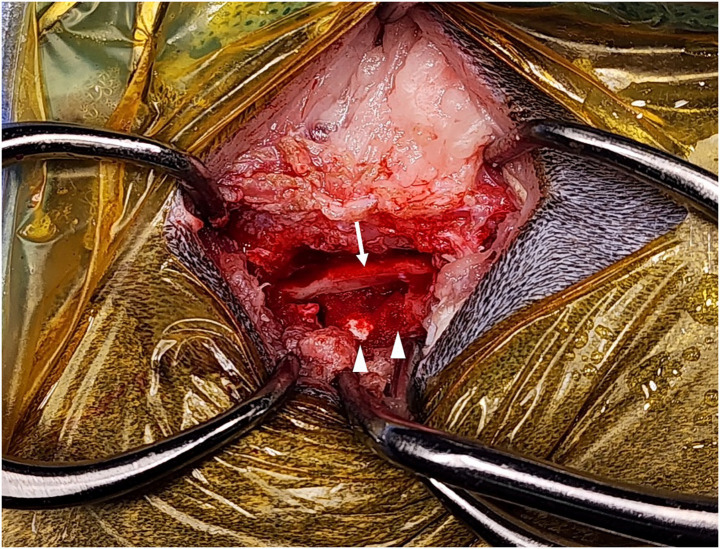
Intraoperative view following pediculectomy and corpectomy at the T11 vertebra. The decompressed spinal cord is visible through the surgical window (arrow). An absorbable gelatin sponge was applied to control intraoperative bleeding at the corpectomy site (arrowhead).

Histopathology was performed independently at two laboratories (Antech Diagnostics, Fountain Valley, CA, United States; IDEXX Laboratories, Westbrook, ME, United States) using separate tissue sections. In one laboratory, the lesion was described as a poorly demarcated mass lesion expanding into the marrow spaces and separating the adjacent bone (Figures 4A,B). The lesion comprised a disorganized vascular proliferation characterized by tangles of capillaries lined by smooth muscle and supported by a loose collagenous stroma. The endothelial cells were plump, with oval nuclei, condensed chromatin, one to two small nucleoli, scant cytoplasm and indistinct cell borders. The bone lining the lesion comprised woven trabeculae, each bordered by a single layer of osteoblasts, and occasional osteoclasts were observed. These findings are consistent with an AVF with osseous proliferation and bone remodeling. In the other laboratory, the samples consisted of irregular fragments of variably mature bone with multifocally irregular scalloped margins, resorption lines, and surface deposits of reactive woven bone (Figures 4C,D). The interceding tissue consisted of collagenous stroma that was loose and edematous, with markedly increased numbers of innumerable haphazardly arranged capillary-type blood vessels lined by plump to minimally atypical endothelial cells. Endothelial cells had round to oval nuclei with finely stippled chromatin and 1–2 small to punctate nucleoli. The overall histologic features were most suggestive of a capillary-type vertebral angiomatosis.

**Figure 4:**
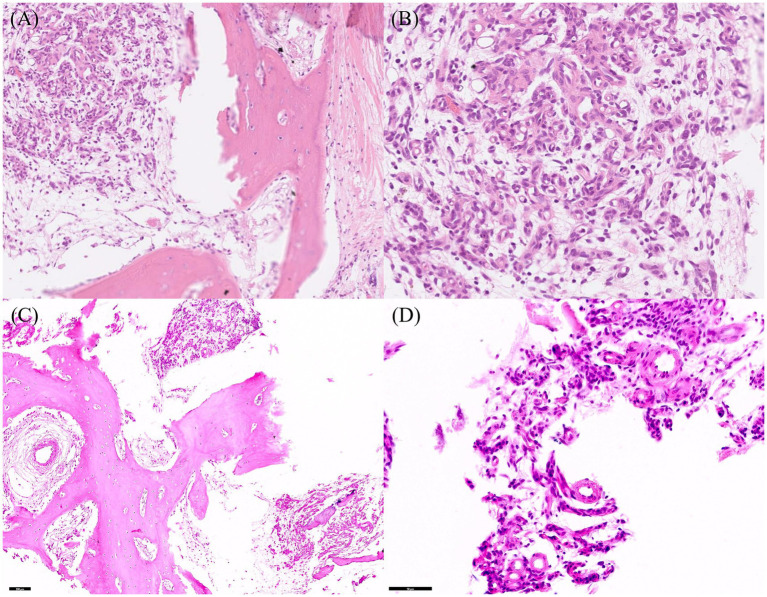
Histopathologic sections of spinal lesions at T11 in a cat (H&E). **(A,B)** Sections from an extradural mass demonstrating disorganized vascular proliferation with tangles of capillaries within a loose collagenous stroma, accompanied by woven bone lined by osteoblasts and occasional osteoclasts, consistent with an arteriovenous fistula with associated osseous proliferation and bone remodeling. **(A)** Original magnification ×20. **(B)** Original magnification ×40. **(C,D)** Sections from additional tissue samples showing irregular fragments of variably mature bone with reactive woven bone and resorption lines, accompanied by marked proliferation of haphazardly arranged capillary-type blood vessels within a loose, edematous, collagenous stroma. Endothelial cells are plump but minimally atypical, with round to oval nuclei and finely stippled chromatin, and no mitotic figures are identified. These features are most suggestive of capillary-type vertebral angiomatosis. **(C)** Original magnification ×10. **(D)** Original magnification ×40.

By postoperative day 7, the cat had improved from non-ambulatory paraparesis to ambulatory paraparesis. By day 20, normal gait had returned, and urinary and fecal continence were fully restored. No recurrence of clinical signs was observed up to day 75 postoperatively, after which follow-up was discontinued.

## 3 Discussion

Vascular malformations refer to congenital quantitative or structural abnormalities of arteries, veins, capillaries, and lymphatic vessels (17). In the human central nervous system, non-neoplastic vascular malformations include arteriovenous malformations, cavernous angiomas, venous angiomas, and capillary telangiectases (25, 26). Among these, an arteriovenous shunt (also referred to as arteriovenous malformation or fistula) is defined as abnormal connections between arteries and veins that bypass the normal capillary bed (13). On the other hand, angiomatosis has been described using various terms, including hemangiomatosis, hamartoma and lymphangiomatosis (27), and is generally regarded as a benign vascular proliferation (12).

SAVF and vertebral angiomatosis are rare vascular lesions reported in cats. They are commonly described in young animals between 1 and 2 years of age, typically involving the thoracic vertebrae, and share many clinical features, including pain, gait abnormalities, and urinary or fecal dysfunction (1–10).

Previously reported cases of SAVF in cats have demonstrated dilation of the internal vertebral venous plexus, intercostal veins, interspinous veins, azygos vein, and interarcuate branch on CT angiography or MRI, and early contrast enhancement of these venous structures during the arterial phase was reported in those cases (10). In contrast, the imaging findings in the present case differed from those in previously reported feline SAVF cases (10). No arteriovenous shunt or dilated venous structures were identified. Instead, a well-defined osseous proliferation centered on the T11 pedicle and vertebral body was observed, resulting in extradural spinal cord compression.

When vertebral proliferation is observed in the absence of overt vascular abnormalities, the differential diagnoses commonly include primary bone tumors, bone infection, reactive callus formation, and cartilaginous exostoses (2, 28, 29). However, in the present case, these differentials were considered less likely given the patient’s young age, lack of traumatic history, and the solitary nature of the lesion. In contrast, previously reported cases of feline vertebral angiomatosis have been characterized by thickening of the vertebral body extending to the lamina and pedicle, often leading to extradural spinal cord compression (1–9). In this respect, the imaging features of the present case were more similar to vertebral angiomatosis than to the other differential diagnoses considered.

Interestingly, a canine case of SAVF has also been reported with a mass-like vertebral lesion that required differentiation from vertebral angiomatosis (19), and in human medicine, intraosseous AVF has likewise been reported to resemble vertebral angiomatosis on imaging studies (20–22). Accordingly, the present case demonstrated imaging features resembling those of vertebral angiomatosis while simultaneously sharing certain characteristics with previously reported SAVF cases.

In addition, histopathologic features of both lesions were observed concurrently in this case. In the first histopathologic evaluation, the lesion was interpreted as an AVF accompanied by osseous proliferation and bone remodeling; however, a second evaluation was most consistent with capillary-type vertebral angiomatosis. These different interpretations may not represent conflicting diagnoses but rather reflect variation in the relative prominence of specific structural components within a vascular malformation, depending on the histopathologic section examined.

Indeed, a human pathomorphologic study reported the presence of arteriovenous micro-shunts in small-vessel hemangiomas of arterial or venous origin (17). However, hemangiomas composed purely of capillary elements are generally not associated with an arteriovenous shunt (17). Therefore, the concurrent SAVF and capillary-type angiomatosis in the present case may initially appear contradictory. However, in other sections, a tangled network of small capillaries and arteriolar-sized vessels was observed, along with larger vessels containing an internal elastic lamina transitioning into venous structures. These findings suggest that, rather than representing a purely capillary lesion, the relative predominance of arterial, venous, and capillary components may have varied depending on the histologic section examined.

Although cases in which SAVF and vertebral angiomatosis are definitively confirmed concurrently are rare, a human report has documented grossly identifiable connections between these two lesions (30). In that report, intraoperative manipulation of blood flow resulted in observable changes in the lesion, suggesting hemodynamic interaction between the two lesions. Such observations indicate that AVF and hemangioma may not be entirely distinct pathologies but may influence one another through local hemodynamic changes. Therefore, the capillary proliferation interpreted as hemangioma in the present case may have been secondary to the underlying AVF.

When SAVF and vertebral angiomatosis share certain histopathologic and imaging features, the key consideration is not strict differentiation between the two conditions but identification of the predominant pathophysiologic mechanism contributing to the neurologic deficits. In vertebral angiomatosis, spinal cord injury is generally attributed to extradural compression secondary to vertebral proliferation (1–9). In contrast, SAVF has a more complex pathophysiology, as described primarily in human spinal arteriovenous shunt literature. Direct arteriovenous shunts allow arterial blood to enter the venous system, resulting in venous arterialization and venous hypertension (14, 15). Elevated venous pressure leads to venous congestion and dilation, which may cause direct spinal cord compression (13, 31). This congestion may reduce the arteriovenous pressure gradient within the spinal cord, impairing tissue perfusion and leading to progressive neural hypoxia and intramedullary edema (15). In addition, diversion of blood flow from the capillary bed can produce a vascular steal phenomenon, reducing spinal cord perfusion and worsening ischemic injury (32).

Several human cases of spinal intraosseous AVF have been reported to present as expansile vertebral lesions mimicking bone tumors or vertebral angiomatosis on imaging studies (20–22). In a canine case of SAVF, vertebral hyperostosis and remodeling were considered likely secondary to chronic hypoxic bone damage and contributed to spinal canal stenosis and secondary spinal cord compression (19). In the present case, no dilated venous structures, intramedullary edema, or definite arteriovenous shunt was clearly identified on imaging. Instead, the dominant imaging abnormality was an expansile osseous lesion causing extradural spinal cord compression. Therefore, the neurological dysfunction was considered more likely attributable to mechanical compression by the proliferative vertebral lesion, more closely resembling the mechanism described in feline vertebral angiomatosis (1–9).

These pathophysiologic differences have important therapeutic implications. When venous hypertension and vascular steal predominate, endovascular embolization or surgical ligation of the spinal arteriovenous shunt may represent a primary treatment strategy in human medicine (32, 33). Although these approaches have not been well documented in veterinary SAVF cases, they have been discussed as potential therapeutic options in previous reports (19). In contrast, when mass effect is the primary mechanism, as in the present case, surgical decompression becomes the main therapeutic approach for compressive spinal cord lesions in small animals (34). Histopathological examination is essential for definitive tissue diagnosis and may also provide prognostic information. Imaging evaluation serves a complementary role by defining lesion distribution, spatial extent, effects on adjacent structures, and, importantly, the predominant pathophysiologic mechanism responsible for neurological dysfunction. Together, these modalities provide a more complete basis for diagnosis and therapeutic decision-making.

In this case, only conventional contrast-enhanced CT and MRI were performed, without further evaluation of the arterial and venous phases via CT angiography (CTA) or MR angiography (MRA). In human medicine, selective digital subtraction angiography is considered the gold standard for diagnosing arteriovenous shunts (35). In veterinary medicine, CTA and MRA have increasingly been used for this purpose (10, 19, 36). These modalities achieve higher diagnostic sensitivity for SAVFs than conventional CT and MRI by enabling both evaluation of the arterial and venous phases and precise localization of the feeding arteries and draining veins, thus providing information that is essential for therapeutic planning (37). Here, the absence of angiographic evaluation limited the ability to accurately determine the location and type of SAVF.

In conclusion, the present case demonstrates that SAVF and vertebral angiomatosis may overlap both histologically and in their imaging findings. Diagnostic imaging should serve not merely to distinguish between these two lesions but to ensure that vascular malformation is considered in the differential diagnosis of expansile vertebral lesions in young cats. Furthermore, diagnostic imaging evaluation helps identify how a lesion is expressed within the vascular malformation spectrum and the associated predominant pathophysiologic mechanism underlying the neurologic deficit, which is critical for therapeutic decision-making.

## Data Availability

The original contributions presented in the study are included in the article/Supplementary material, further inquiries can be directed to the corresponding author.
